# Psychometric evaluation of the perceived perioperative competence scale-revised among the Chinese operating room nurses: a methodological research

**DOI:** 10.1186/s12912-022-00853-x

**Published:** 2022-04-07

**Authors:** Qiaomeng Yu, Ran Wei, Yongting Wei, Xiuhong Wu, Tao Liang

**Affiliations:** 1 Department of Operating Room, National Cancer Center/National Clinical Research Center for Cancer/Cancer Hospital, Chinese Academy of Medical Sciences and Peking Union Medical College, Beijing, 100021 China; 2 Department of Radiation Oncology, National Cancer Center/National Clinical Research Center for Cancer/Cancer Hospital, Chinese Academy of Medical Sciences and Peking Union Medical College, Beijing, 100021 China; 3 National Cancer Center/National Clinical Research Center for Cancer/Cancer Hospital, Chinese Academy of Medical Sciences and Peking Union Medical College, Beijing, 100021 China; 4 School of Nursing, Peking Union Medical College, Beijing, 100730 China

**Keywords:** Operating room nurse, Perioperative competence, Scale, Psychometric, Validity

## Abstract

**Background:**

Perioperative competence is necessary to evaluate operating room nurses. The Perceived Perioperative Competence Scale-Revised (PPCS-R) is the only available tool developed specifically for the perioperative setting. However, there is a lack of research on the reliability and validity of this scale among Chinese nurses. Thus, the aim of this study is to translate, culturally adapt, and evaluate the psychometric properties of the Perioperative competence Scale-Revised (PPCS-R) among operating room nurses in China.

**Methods:**

Instrument cultural adaptation was carried out through forward translation, back translation, expert panel evaluation and pretesting. The psychometric properties (content validation, item analysis, construct validation, and reliability coefficient) of the Chinese PPCS-R (C-PPCS-R) were examined. An online survey was completed from June to August 2020 by operating room nurses (*N* = 480) in five third-grade class-A hospitals in Beijing.

**Results:**

The item analysis identified six items for scale reduction. Exploratory factor analysis showed the remaining 34 items loaded on six factors, which were named in accordance with the original scale. The six-factor model showed a good fit through confirmatory factor analysis. The item content validity index for the C-PPCS-R items ranged from 0.857 to 1.000, and that for the total scale was 0.875. Cronbach’s alpha was showed 0.787. Together, the six factors explained 68.62% of the variance.

**Conclusions:**

The 34-item C-PPCS-R showed good validity and reliability to measure perceived competence among operating room nurses in the Chinese context. The scale can assist nurse managers to identify operating room nurses’ perceived competence, and provides evaluation criteria for career planning, performance appraisal, job assignment, and continuing education.

## Introduction

Nurses have an important role in promoting patients’ health. As their contribution is indispensable, it is essential that they can perform their job optimally. Competence is a crucial attribute for excellent work performance. It refers to an individual’s deep-seated characteristics and can be used to distinguish outstanding performers from others in a job. Competence encompasses incentive, self-image, individual traits, knowledge of a certain field, attitudes or values, and cognitive or behavioral abilities [1]. Over the past decade, this concept has been applied in nursing management studies, and is defined as the performance of nursing roles to required standards in three aspects: knowledge, understanding, and judgment; a range of cognitive, technical/psychomotor, and interpersonal skills; and a range of personal attributes and attitudes [2].

Competence has been conceptualized in several frameworks. For example, Benner considered seven aspects of competence: helping role, teaching counseling, diagnosis function, management situation, treatment intervention, quality assurance, and job role [3]. Benner’s theory suggests that clinical nurses undergo five developmental stages from novice to expert: novice, advanced novice, competent nurse, proficient nurse, and expert. Other guidelines also give some indicators of competences for nurses. The International Council of Nurses (ICN) developed the ICN Framework of Competences for Generalist Nurses in 2003, which were updated to reflect evolving practice in 2008 [2, 4].

Different generic instruments have been developed to measure perceived competence in nursing based on various theoretical frameworks. Some examples are: the Nurse Competence Scale, which has 73 items on seven subscales [5]; the Holistic Nursing Competence Scale, which has 36 items on five subscales [6]; the Competency Inventory for Registered Nurses Scale, with 58 items on seven subscales [7, 8]; and the Australian Nursing Competency Incorporated 2000 standards, with 51 items across four subscales [9]. The measurement properties of these scales have been investigated, and the scales were found to be well-validated in their original languages and contexts [10]. Some scales have been used to measure the competence of nurses in different hospital working environments, such as wards, emergency departments, intensive care units, or operating rooms [11, 12].

Regular assessment of the competences of practicing nurses can help improve the quality of nursing, and provision of ethical and safe nursing care. However, a major weakness in the usage of generic tools is their failure to capture contextual nuances that distinguish clinical practice in specialized fields. Perioperative nursing is a sub-specialty nursing area that is independent from other medical settings, and is considered one of the most potentially hazardous of all clinical environments. It requires more comprehensive knowledge about surgical coordination, management and use of surgical instruments and equipment, anesthesia care, as well as intraoperative resuscitation and staff deployment. In addition to the surgeon, the patient is another important person that the operating room nurse directly serves. During surgery, when the patient is under anesthesia, the operating room nurse has a more important role to play in supervising and ensuring the safety of the patient during the operation. Therefore, from the patient’s point of view, there is also a need for nurses who are more competent to work in the operating room to take responsibility for their life safety [13]. Pre- licensure education programs seldom contain any meaningful perioperative nursing component. In addition, practice in an unfamiliar subspecialty may mean nurses temporarily revert to a lower level of competence [14]. However, an appropriate self-assessment measurement was scarce for testing nurses’ perceived competence in the operating room setting in China. Therefore, it is important to investigate an appropriate instrument to provide evaluation criteria for career planning and promotion evaluation among operating room nurses.

## Background

### Perioperative nursing competence

It’s necessary for perioperative nurses to grasp the requirements for clinical and service quality, coordinated patient-centered care, information management, efficiency, cost-effectiveness, and the importance of patient satisfaction to prepare and arrange themselves for their career [15]. Previously, “operating room (“OR”) nurse” was specifically defined as caring that perform during the intraoperative period, but the scope has broadened with the role of perioperative nurse [16]. There are diverse perioperative nursing roles, including scrub nurses, circulating nurses, registered nurse first assistants, and advanced practice registered nurses, as well as emerging roles such as robotics coordinators and informatics specialists [15].

Perioperative/operating room nurses should be oriented to both the circulating and the scrub roles during their orientation period. A circulating nurse uses professional judgment to direct, manage, and delegate nursing aspects of care throughout the perioperative phase [17]. A scrub nurse works directly with the surgeon within the sterile field, and this role involves a detailed understanding of each phase of the surgical process and the ability to predict and supply equipment as appropriate [17]. Infection control is also an important competence of perioperative nurses. Because the operating room surface poses a risk to both patients and personnel, environmental sanitation and terminal cleaning are critical to preventing the transmission of disease. When possible, keep clean and polluted areas physically segregated. For infectious patients who also require emergency surgery, personnel must follow preventative steps. In the operating room, personnel must use a N95 respirator without exhalation valves, as recommended by the National Institute for Occupational Safety and Health [17]. As a result, the perioperative nurse is gradually replacing the operating room nurse as the internationally recognized and more comprehensive term for nurses working in the operating room setting. They usually refer to the same group [18].

Under the perioperative setting, nurses serve as a conduit between the patient and the machineries used. Nurses provide a deeper sense of and compassion for the patient on the level of social psychology [19]. Teamwork and communication, collaboration, clinical leadership, and coordination have been related to improvement of competence during the perioperative environment. These skills encompass the ability to resolve conflict, prioritize, and organize human and material resources according to changing and frequently uncertain demands in the clinical community [19].

The initial stage of developing an assessment tool requires a theoretical framework based on qualitative research. The majority of previous studies used a qualitative approach to explore the development of a perioperative nursing competence system or guideline standards. These theoretical frameworks included several aspects: 1) professional basic skills, 2) interpersonal skills, 3) career development ability, and 4) individual characteristics [20–24]. To date, the Perceived Perioperative Competence Scale-Revised (PPCS-R) is the only instrument specifically for the perioperative setting that has been rigorously developed and psychometrically validated. Perioperative nursing competences can be categorized as technical or nontechnical competences. Technical competences are related to situational and practical knowledge, etiquette knowledge and practice standards. Non-technical abilities are associated with empathy, integral caring, communication, collaboration and teamwork [25].

### Development and application of the PPCS-R

The initial creation of the PPCS-R was guided by an integrated literature review, three earlier researches [26–28], an improved Delphi panel evaluation, and a pilot investigation [29]. This process resulted in development of a 94-item scale. The instrument was tested using a national sample comprising 3209 nurses who belong to the organization of Australian College of Operating Room Nurses. Utilizing project analysis, exploratory factor analysis (EFA), and the Cronbach’s alpha coefficients, the 94-item scale was reduced to a final PPCS-R with 40 items on six factors. These factors were: Foundational knowledge and skills, Leadership, Collaboration, Proficiency, Empathy, and Professional development. The value of Cronbach’s alpha for the 40-item scale was 0.96 and the six factors contributed for 58.6% of the overall variance. The answer to the 40-item PPCS-R is on a five-point Likert scale ranging from never to always (1 to 5). Total scores are possible between 40 and 200, higher scores suggesting greater perceived competence levels [25].

The measurement properties of the scale were also widely analyzed and found to be well-validated in a number of countries. A study aimed to compare distinctions in perceptions of competence among nurses and perioperative technicians in the Canadian and Scottish contexts [30]. The primary research carried out in Sweden aimed to assess PPCS-R using the confirmatory factor analysis (CFA) in Swedish, and compare competence between operating room nurses and registered nurse anesthetists [31]. A study conducted in Turkey completed a methodological design of the Turkish adaptation for the PPCS-R and carried out a psychometric evaluation of the scale for use in that context [32]. An Iranian study that evaluated the psychometric properties of the Persian version of the instrument reported the psychometric testing showed good results. The Persian version was a rigorous five-subscale instrument suitable to assess the perceived perioperative competence level of Iranian operating room students [33].

Chinese OR nurses are essentially the same as foreign OR nurses in terms of competence, but there are some cultural differences in expression for the measurement instruments, so corrections need to be made to modify the entries to fit the Chinese context. Thus, the aim of the current research was to report the Chinese adaptation and psychometric evaluation of the PPCS-R among the OR nurses.

## Methods

This adaptation and evaluation of the PPCS-R involved two phases. Phase one comprised the translation, adaptation, and content validation of the PPCS-R items. Phase two consisted of collecting data from operating room nurses using the newly adapted 40-item C-PPCS-R and evaluating the psychometric properties of the instrument. Phase two included three sections, items analysis, construct validity and Reliability analysis. Items analysis used through discrete trend analysis, factor analysis, the critical ratio (CR) method, and the correlation coefficient method. Data were analyzed using the construct validity index, EFA and CFA. Reliability analysis was performed using Cronbach’s alpha.

### Participants and setting

Based on the theory that the sample size should be between 5 to 10 times the amount of scale items for factor analysis [34], it was thought that 200–400 participants would be an ideal sample size because 40 items would be involved in this study. The minimum sample size was doubled to at least 400 nurses because we expected to conduct both EFA and CFA. The present study was conducted from June to August in 2020, and included 480 operating room nurses who worked in five third-grade class-A hospitals (highest-level hospitals) in Beijing. We used random sampling to divide participants into two groups: Group A (*n* = 240) for item analysis and EFA, and Group B (*n* = 240) for CFA. Participants were eligible for this study if they: were registered nurses in China; had at least 1 year of working experience in the operating room (The teaching period for new nurses is 1 year); and were willing to participate in this study voluntarily. Nurses who were on long-term sick or pregnancy leave or that were informal employees were excluded. All participants will henceforth be referred to as operating room nurses.

### Phase one: development of the 40-item Chinese PPCS-R

To adapt the scale to Chinese culture, the PPCS-R was first translated from English to Chinese using forward-translation. This was followed by back-translation into English to evaluate the content validation.

### Step 1: translation and cultural adaption

Five Chinese researchers translated the instrument. The initial scale was translated from English to Chinese utilizing forward-translation independently through a postgraduate nurse with excellent English translation skills and an undergraduate operating room nurse from our research team. Next, another postgraduate nurse with rich experience in operating room nursing and the two translators from the previous step discussed and examined any unclear passages in these two editions together.

Following this discussion, two bilingual experts were invited to review the initial Chinese version of the scale and translate it back into English independently. These two experts majored in English and had worked in English-speaking countries for some years. Based on discussions with these experts, we made several revisions to the scale to ensure equivalent semantics. The 40-item Chinese version (C-PPCS-R40) was developed after changing five items (see Table 1 for details of these modifications and the underlying reasons).

**Table 1:** Items that were changed and the reasons for modification

No.	Before	After	Reason
1	I am familiar with most of the instrumentation in different specialties.	I am familiar with most of the instrumentation in different sub-specialties medical subjects. (e.g. neurosurgery, orthopedics)	The concept of specialty in Chinese OR context usually use the term “sub-specialty” to describe and give some examples.
5	I am familiar with the technological equipment used in the OR.	I am familiar with all kinds of regular technological equipment in the OR. (e.g. electrosurgery, operating lamp, operating table)	Giving some examples of the regular technological equipment to make the item clearer
35	I maintain current knowledge of, and incorporate relevant organizational policies into practice.	I understand relevant organizational policies and could put them into practice. (e.g. Medical insurance policy, Charging standards.)	Especially emphasize the understanding of organizational policies and give some examples.
37	I maintain knowledge of, and incorporate relevant standards into my practice.	I understand and could work based on the standard of all relevant professional official guidance on practice.	Especially emphasize the standards that mean the guidance or consensus in professional areas.
39	I keep up with the technical changes in procedures and equipment.	I keep up with the technical changes in procedures and equipment. (e.g. Application of intelligent OR, Robotic surgical instrument management and operation cooperation, Magnetic navigation surgical coordination)	Giving some examples of the technical changes in procedures and equipment to make them clearer.

### Step2: content validation

A content validity questionnaire was created to evaluate the understandability of the C-PPCS-R40 and the correlation. On a four-point Likert scale, the questionnaire items were graded from 1 (‘not relevant’) to 4 (‘very relevant’) [35]. A panel of seven perioperative nurse experts with more than 20 of years working experience that served as operating room leaders in third-grade class-A hospitals in Beijing was invited to complete this questionnaire. Each panel member completed the questionnaire independently. The item content validity index (CVI) for each C-PPCS-R40 item was from 0.857 to 1.000 and the total scale CVI was 0.875 (Table 2).

**Table 2:** C-PPCS-R40 and the content validity index for each item

Item	I-CVI
Q1. I am familiar with most of the instrumentation in different sub-specialties medical subjects. (e.g. neurosurgery, orthopedics)	1
Q2. I know where to find equipment and supplies in the OR.	1
Q3. My local knowledge of this department assists me to perform my OR role.	1
Q4. I understand and anticipate the surgical procedure.	1
Q5. I am familiar with all kinds of regular technological equipment in the OR. (e.g. electrosurgery, operating lamp, operating table)	1
Q6. When I am allocated to an area of the OR that is unfamiliar, I draw on my skills and experience.	1
Q7. I plan and coordinate the needs in the theatre I am allocated.	1
Q8. I know instinctively when surgery is not going well and am able to respond appropriately.	1
Q9. Knowing the location of equipment in the OR assists me to perform my OR role.	1
Q10. I take a leadership role to ensure the smooth running of the theatre.	1
Q11. I make difficult decisions when necessary.	0.857
Q12. I take an active role in preceptoring or mentoring lesser experienced nurses.	1
Q13. I manage clinical situations when there is conflict between staff	0.857
Q14. I provide clinical guidance to other staff members.	1
Q15. I encourage team members to use innovative solutions to solve traditional problems.	1
Q16. I delegate aspects of care according to role, functions, capabilities and learning needs of other team members.	1
Q17.I encourage active involvement in clinical decision-making processes.	1
Q18. I use appropriate methods of communication according to the needs of the situation.	1
Q19. I feel comfortable in seeking assistance from my colleagues when I am unsure.	1
Q20. I tailor my communication based on the mix of personalities in the team.	1
Q21. I respect the level of expertise of other members of the team.	1
Q22. I treat members as individuals who have different needs, abilities and aspirations.	0.857
Q23. When communicating with other team members, I use language that is appropriate to the situation.	1
Q24. I have mastered the terminology and vocabulary of OR nursing.	1
Q25. I troubleshoot and take appropriate action in the event of machine / equipment failures.	1
Q26.Based on experience, I am able to identify actual or potential emergency situations and respond appropriately.	1
Q27. I apply specialist knowledge in providing care for OR patients.	1
Q28. I have the right amount of knowledge to practice in this specialty.	1
Q29. I am able to anticipate the needs of the situation.	1
Q30. I consider my colleagues as individuals with different needs, abilities and aspirations.	1
Q31. I am willing to seek help from colleagues when I am uncertain about the work.	1
Q32. I adjust my way of communication according the character of my colleagues.	1
Q33. When communicating with other team members, I could use language that is appropriate in that context.	1
Q34. I establish rapport with patients that enhances their ability to express feelings and concerns.	0.857
Q35. I understand relevant organizational policies and could put them into practice. (e.g. Medical insurance policy, Charging standards.)	1
Q36. I have detailed knowledge of anatomy and physiology.	1
Q37. I understand and could work based on the standard of all relevant professional official guidance on practice.	1
Q38. I read current journals and literature that relate to clinical practice.	1
Q39. I keep up with the technical changes in procedures and equipment. (e.g. Application of intelligent OR, Robotic surgical instrument management and operation cooperation, Magnetic navigation surgical coordination)	0.857
Q40. I use available resources to maintain current OR practice.	1

### Step 3: pretesting

Next, 12 operating room nurses from the Cancer Hospital Chinese Academy of Medical Sciences were invited to evaluate the feasibility and understandability of the C-PPCS-R40. These nurses were recruited based on their level of seniority, which was divided into four levels: ≤3 years, 4–10 years, 11–15 years, and ≥ 16 years. Each level was represented by three nurses. The nurses provided feedback independently regarding the understandability of the C-PPCS-R40, and indicated the meaning of the items was clear.

### Phase two: psychometric evaluation of the C-PPCS-R40

Phase two included item analysis, construct validation test, and reliability analysis of the C-PPCS-R40.

### Item analysis

The items were selected through discrete trend analysis, factor analysis, the CR method, and the correlation coefficient method. The details of these analyses are as follows. Discrete trend analysis was conducted to calculate the coefficient of variation (CV) of each item, and items with CV ≤0.20 were deleted. Items with communalities greater than 0.20 and factor loading greater than 0.45 were retained. The CR method was used to rank the total scores for the scale; the 27% of the sample with the highest and lowest scores were identified, and an independent samples t-test was used to compare the scores between the two groups (any items that were not significant at p>0.05 or |t|<3 were deleted). The correlation coefficient was calculated and items with a correlation < 0.30 between the score for that item and the total score of the scale were removed. In a comprehensive discussion, under the condition that the scale structure is not seriously affected, the items that meet two or more deletion conditions simultaneously are considered to be discarded. Each factor should contain no less than three items [34].

### Construct validation test and reliability analysis

EFA was carried out to determine the dimensionality and factor structure of the C-PPCS-R40 after item analysis. To determine the appropriateness of the factor model and sample size for the factor analysis, the Kaiser-Meyer-Olkin (KMO) value and Bartlett’s sphericity test were used. A KMO value no less than 0.80 shows an appropriate sample size and the statistical significance of Bartlett’s sphericity test (at the level of 0.05) shows suitability for a factor model. Varimax rotation was applied and items were selected based on eigenvalues > 1.0 and estimates of minimum factor loading of 0.40. CFA was intended to validate if the C-PPCS-R40 factors were appropriate. Model fit indexes were measured according to the χ^2^/df ratio, standardized root mean square residual (SRMR), root mean square error of approximation (RMSEA), Tucker–Lewis index (TLI), and comparative fit index (CFI). If the χ^2^/df ratio was < 3, SRMR and RMSEA values were ≤ 0.08, and TLI and CFI values were ≥ 0.90, the model fit was deemed to be acceptable. Item internal consistency and the homogeneity of the C-PPCS-R40 were evaluated using Cronbach’s alpha. The values of Cronbach’s alpha coefficient ≥ 0.90 demonstrate excellent internal consistency, 0.80–0.90 good internal consistency, and 0.70–0.80 acceptable internal consistency [36, 37].

### Consideration of ethics

Permission from the scale creators was received to translate and use the original scale. The leaders of the operating rooms in the five participating third-grade class-A hospitals granted permission for nurses to complete the scale online. Participants from the five hospitals were assured of their anonymity and the voluntary nature of participation. Participants’ consent was implied by the return of the survey form. All methods were carried out in accordance with relevant guidelines and regulations. The research proposal for this study involving human participants were approved by the ethics committee of National Cancer Center/Cancer Hospital, Chinese Academy of Medical Sciences.

### Data collection

A web survey convenience sampling method used to collect data from participants, which included the C-PPCS-R40 and a demographic questionnaire created by the present investigators. The five hospitals OR directors assisted the researchers in recruiting eligible OR nurses. Eligible and voluntary OR nurses participated in the study by joining a web group (WeChat) where the researcher posted a link to the web questionnaire. The web questionnaire was edited by an authoritative and confidential questionnaire platform, and the results of the information were available only to the researchers. Uniform instructions and instructions for completing the questionnaire in which the investigator spells out in detail the purpose of the study and the exclusion criteria for inclusion. OR nurses who participated in the questionnaire and responded after reading the instructions were considered to have voluntarily participated in the study. Participants have the right to withdraw from this study at any time. Datum were obtained from June to August, 2020.

### Data analysis

SPSS version 25 and AMOS version 23 were taken advantage of the statistical analysis. To investigate the characteristics of the sample using descriptive analysis. In order to rotate the factors while preserving independence, EFA was undertaken using orthogonal rotation (varimax) and eigenvalues greater than 1.0. The suitability of the datum for factor analysis was calculated using the KMO sampling adequacy measure and the Bartlett’s sphericity test. For group variables, a minimum loading approximation of 0.40 was used. AMOS version 23 was used to conduct CFA to test the construct validation of the C-PPCS-R40. Cut-off values and goodness of fit indexes were used to determine if the model was suited to the datum logically. The internal consistency and homogeneity of the C-PPCS-R40 were evaluated using Cronbach’s alpha [36, 37].

## Results

### Participants

In total, 521 OR nurses joined the network group, 505 of them completed the C-PPCS-R40 scale and demographic questionnaire. If the same answer appeared in 10 consecutive items, that response was considered invalid. The final number of effective responses was 480. The majority of the sample was female (85.42%), the mean age was 30.64 ± 6.61 years, and the mean number of years of operating room nursing experience was 9.01 ± 6.91 years. More than half of the participating nurses had a bachelor’s degree or above (60.42%) and the most common professional rank was “primary nursing” (66.87%). Most nurses were involved in clinical work (84.17%), and few were involved in research (2.71%). Table 3 presented further information about the sample.

**Table 3:** Characteristics of operating room nurses in the sample (*n* = 480)

Characteristic	*n*	%	M	SD
Gender				
Male	70	14.58		
Female	410	85.42		
Age (year-old)			30.64	6.61
≤ 25	130	27.08		
26 ~ 35	244	50.83		
36 ~ 45	86	17.92		
≥ 46	20	4.17		
Years of OR experience			9.01	6.91
≤ 3	140	29.17		
4 ~ 10	177	36.87		
11 ~ 15	85	17.71		
≥ 16	78	16.25		
Highest education				
Junior college Diploma or Below	190	39.58		
Bachelor or Over	290	60.42		
Experience of on-the-job education				
Yes	279	58.12		
No	201	41.88		
The professional rank				
Primary nursing	321	66.87		
Secondary nursing	156	32.50		
Advanced nursing	3	0.63		
Primary role in OR (Multiple choices)				
Clinical nurse (Yes/No)	404/76	84.17/15.83		
Nurse educator (Yes/No)	104/376	21.67/78.33		
Nurse manager (Yes/No)	45/435	9.37/90.63		
Nurse researcher (Yes/No)	13/467	2.71/97.29		
Obtained OR specialty education				
Yes	293	61.04		
No	187	38.96		

### Item analysis

All communalities values (C^2^) were greater than 0.20 and factor loadings were greater than 0.45. The coefficients of variation (CV) for item 9, 11, 23, 27, 29 were ≤ 0.20. The item-total correlations for 19 items were < 0.30. Item 9, 11, 23, 24, 25, 26, 27, 29, 33, 34, 35 and 40 were not significant (*p*>0.05) or |t|<3 according to the CR results. The item 9, 11, 23, 27 and 29 that meet three deletion conditions simultaneously were given priority to be discarded. While item 24, 25, 26, 33, 34, 35 and 40 faced two deletion criteria, the seven items were the key sections, including Proficiency and Empathy, which, if removed, could influence the structure and sense of the factors of the original scale, six of which could be kept after interviewing experts and only item 40 deleted. Finally, the item analysis identified six items for scale reduction. Table 4 showed further information.

**Table 4:** Item analysis of C-PPCS-R40 (*n* = 240)

Item	Mean ± SD	Coefficient of Variation	Item-total correlation	Critical Ratio (t value)	C^2^	Factor loading
Q1	3.24 ± 0.98	0.30	0.476**	−7.24*	0.860	0.887
Q2	3.25 ± 0.98	0.30	0.420**	−6.33*	0.746	0.838
Q3	3.41 ± 0.97	0.28	0.379**	−5.53*	0.685	0.803
Q4	3.13 ± 0.96	0.30	0.344**	−5.34*	0.677	0.761
Q5	3.33 ± 0.98	0.29	0.439**	−6.92*	0.658	0.796
Q6	3.22 ± 1.24	0.38	0.471**	−7.35*	0.727	0.776
Q7	3.25 ± 1.13	0.35	0.448**	−7.14*	0.642	0.729
Q8	3.23 ± 0.99	0.30	0.446**	−6.53*	0.764	0.818
Q9	3.51 ± 0.50	0.14	0.013	0.34	0.836	0.903
Q10	3.12 ± 1.44	0.46	0.422**	−6.51*	0.803	0.788
Q11	3.53 ± 0.50	0.14	−0.071	1.03	0.390	0.528
Q12	3.38 ± 1.24	0.36	0.416**	−7.20*	0.709	0.824
Q13	3.29 ± 1.25	0.38	0.480**	−7.87*	0.733	0.839
Q14	3.27 ± 1.23	0.38	0.472**	−7.96*	0.702	0.830
Q15	3.16 ± 1.35	0.43	0.505**	−8.81*	0.610	0.756
Q16	3.05 ± 1.49	0.49	0.442**	−7.28*	0.747	0.748
Q17	3.31 ± 1.21	0.37	0.515**	−8.69*	0.827	0.898
Q18	3.42 ± 1.23	0.36	0.328**	−5.34*	0.693	0.710
Q19	3.32 ± 1.18	0.35	0.338**	−5.64*	0.773	0.812
Q20	3.30 ± 1.13	0.34	0.345**	− 5.76*	0.791	0.878
Q21	3.25 ± 1.24	0.38	0.295**	−4.79*	0.759	0.761
Q22	3.47 ± 1.14	0.33	0.317**	−4.49*	0.738	0.796
Q23	3.48 ± 0.50	0.14	0.029	−0.53	0.743	0.853
Q24	2.87 ± 1.21	0.42	0.091	−1.51	0.625	0.779
Q25	2.92 ± 1.18	0.40	0.159*	−2.37*	0.911	0.948
Q26	2.91 ± 1.19	0.41	0.171**	−2.63*	0.866	0.925
Q27	3.42 ± 0.69	0.20	0.072	−0.15	0.559	0.725
Q28	2.79 ± 1.22	0.44	0.200**	−3.25*	0.725	0.839
Q29	3.47 ± 0.63	0.18	−0.002	0.87	0.786	0.871
Q30	3.39 ± 1.05	0.31	0.354**	−5.00*	0.842	0.879
Q31	3.41 ± 1.07	0.31	0.373**	−5.34*	0.845	0.894
Q32	3.29 ± 1.06	0.32	0.209**	−3.42*	0.582	0.605
Q33	3.21 ± 0.99	0.30	0.170**	−2.59*	0.905	0.936
Q34	3.24 ± 1.01	0.31	0.201**	− 2.72*	0.828	0.866
Q35	3.40 ± 1.08	0.32	0.149*	−2.53*	0.848	0.901
Q36	3.41 ± 1.02	0.30	0.209**	−3.62*	0.869	0.923
Q37	3.38 ± 0.99	0.29	0.257**	−4.29*	0.851	0.910
Q38	3.37 ± 1.03	0.30	0.191**	−3.21*	0.837	0.897
Q39	3.42 ± 1.09	0.32	0.230**	−3.64*	0.840	0.887
Q40	3.45 ± 1.11	0.32	0.024	− 0.09	0.586	0.726

C^2^, communalities; CV=SD/Mean

^**^p<0.01

^*^p<0.05

### Construct validation and reliability

#### Exploratory factor analysis

Items for scale reduction were determined on the basis of the result of item analysis in the previous step, which resulted in 34 items being retained in the final scale. Preliminary analyses (KMO = 0.74 and Bartlett’s test of sphericity = 6659.04, *p* < 0.001) indicated these data were suitable for EFA. According to the results of orthogonal rotation by varimax and eigenvalues > 1.0 to rotate the factors while preserving independence, this analysis reported in a six-factor response (Table 5). No cross-loading items were present and 68.62% of the overall variance was accounted for by the six factors, with percentages ranging from 15.52% (Factor 1) to 8.14% (Factor 6).

**Table 5:** Rotated factor matrix for principal component analysis of the C-PPCS-R34 (*n* = 240)

Item	Factor Loadings					
	1	2	3	4	5	6
Q1. I am familiar with most of the instrumentation in different sub-specialties medical subjects. (e.g. neurosurgery, orthopedics)	**0.895**	0.032	−0.027	0.060	−0.036	0.067
Q2. I know where to find equipment and supplies in the OR.	**0.835**	0.004	−0.073	0.016	−0.064	0.071
Q8. I know instinctively when surgery is not going well and am able to respond appropriately.	**0.826**	0.043	−0.021	0.032	−0.014	0.112
Q3. My local knowledge of this department assists me to perform my OR role.	**0.799**	−0.032	− 0.077	− 0.010	−0.116	0.122
Q6. When I am allocated to an area of the OR that is unfamiliar, I draw on my skills and experience.	**0.789**	0.060	0.005	0.073	0.042	0.041
Q5. I am familiar with all kinds of regular technological equipment in the OR. (e.g. electrosurgery, operating lamp, operating table)	**0.788**	0.004	−0.048	0.117	−0.028	0.017
Q4.I understand and anticipate the surgical procedure.	**0.747**	−0.079	− 0.072	0.050	− 0.038	0.041
Q7. I plan and coordinate the needs in the theatre I am allocated.	**0.724**	0.077	−0.059	0.004	0.010	0.051
Q17. I encourage active involvement in clinical decision-making processes.	0.032	**0.893**	0.039	0.054	0.014	−0.037
Q13. I manage clinical situations when there is conflict between staff	0.076	**0.831**	−0.045	−0.026	0.012	0.060
Q14. I provide clinical guidance to other staff members.	0.005	**0.826**	0.071	0.019	−0.004	0.037
Q12. I take an active role in preceptoring or mentoring lesser experienced nurses.	−0.004	**0.819**	−0.064	0.021	0.009	0.018
Q10. I take a leadership role to ensure the smooth running of the theatre.	−0.054	**0.805**	0.043	−0.045	− 0.044	− 0.071
Q16. I delegate aspects of care according to role, functions, capabilities and learning needs of other team members.	−0.020	**0.766**	0.023	−0.032	− 0.003	− 0.026
Q15. I encourage team members to use innovative solutions to solve traditional problems.	0.074	**0.752**	−0.034	0.074	0.054	0.103
Q36. I have detailed knowledge of anatomy and physiology.	−0.048	− 0.050	**0.918**	0.015	−0.061	− 0.004
Q35. I understand relevant organizational policies and could put them into practice. (e.g. Medical insurance policy, Charging standards.)	− 0.056	− 0.080	**0.910**	− 0.011	0.005	− 0.015
Q37. I understand and could work based on the standard of all relevant professional official guidance on practice.	− 0.061	0.052	**0.910**	0.039	−0.065	0.014
Q38. I read current journals and literature that relate to clinical practice.	−0.124	0.043	**0.898**	0.005	−0.089	0.032
Q39. I keep up with the technical changes in procedures and equipment.	−0.061	0.067	**0.898**	−0.008	−0.004	0.032
Q20. I tailor my communication based on the mix of personalities in the team.	−0.007	0.014	−0.035	**0.875**	0.024	0.081
Q19.I feel comfortable in seeking assistance from my colleagues when I am unsure.	0.060	0.012	−0.031	**0.827**	−0.034	0.040
Q22. I treat members as individuals who have different needs, abilities and aspirations.	0.050	0.010	0.012	**0.782**	0.021	−0.030
Q21. I respect the level of expertise of other members of the team.	0.004	0.015	0.039	**0.775**	−0.090	0.057
Q18.I use appropriate methods of communication according to the needs of the situation.	0.169	−0.002	0.044	**0.700**	−0.022	−0.057
Q25. I troubleshoot and take appropriate action in the event of machine / equipment failures.	−0.045	0.032	−0.074	− 0.051	**0.945**	− 0.020
Q26. Based on experience, I am able to identify actual or potential emergency situations and respond appropriately.	−0.038	0.006	−0.031	− 0.012	**0.918**	− 0.061
Q28. I have the right amount of knowledge to practice in this specialty.	−0.035	0.014	−0.009	0.001	**0.842**	0.009
Q24. I have mastered the terminology and vocabulary of OR nursing.	−0.075	− 0.015	− 0.077	−0.044	**0.779**	−0.016
Q34. I establish rapport with patients that enhances their ability to express feelings and concerns.	0.060	0.018	0.039	−0.039	−0.134	**0.747**
Q30.I consider my colleagues as individuals with different needs, abilities and aspirations.	0.141	0.050	0.076	0.162	−0.011	**0.739**
Q32.I adjust my way of communication according the character of my colleagues.	0.048	−0.084	−0.036	− 0.022	0.043	**0.736**
Q33.When communicating with other team members, I could use language that is appropriate in that context.	0.057	−0.016	−0.023	− 0.109	−0.089	**0.736**
Q31.I am willing to seek help from colleagues when I am uncertain about the work.	0.121	0.121	0.006	0.119	0.112	**0.702**

The first factor had an eigenvalue of 15.52%, and included eight items (items 1, 2, 3, 4, 5, 6, 7, 8) with loadings ≥0.40. This factor was named “Foundational knowledge and skills,” consistent with the original 40-item PPCS-R. The second factor, named “Leadership” (consistent with the original scale), comprised seven items (items 10, 12, 13, 14, 15, 16, 17) and accounted for 13.84% of the variance. Factor 3 was named “Collaboration,” and included five items (items 18, 19, 20, 21, 22) with high loadings and accounted for 12.29% of the variance. Two items were deleted (items 27 and 29) from the original subscale and the remaining four items (items 24, 25, 26, 28) were named “Proficiency” (Factor 4). This factor accounted for 9.57% of the variance. Factor 5 had five items (items 30, 31, 32, 33, 34) with high loadings, and accounted for 9.26% of the variance; this factor was named “Empathy,” and was consistent with the item numbers and factor name of the original scale. The five items (items 35, 36, 37, 38, 39) that were heavily loaded on Factor 6 contributed to the attempts of nurses to keep professionally latest. This was also consistent with the original subscale and named “Professional Development.” Factor 6 accounted for 8.14% of the variance, and only one item (item 40) in this subscale was deleted from the original PPCS-R (Table 6).

**Table 6:** Results of factor extraction by principal component analysis and the Cronbach’s alpha coefficients for the C-PPCS-R34 and each subscale

Subscale	Proportion (%)	Cronbach’s alpha coefficient
Foundational Knowledge and Skills (Item1,2,3,4,5,6,7,8)	15.52	0.921
Leadership (Item10,12,13,14,15,16,17)	13.84	0.913
Collaboration (Item18,19,20,21,22)	12.29	0.855
Proficiency (Item24,25,26,28)	9.57	0.899
Empathy (Item30,31,32,33,34)	9.26	0.792
Professional Development (Item35,36,37,38,39)	8.14	0.949
Total Scale	68.62	0.787

The above analysis resulted in the 34-item Chinese Perceived Perioperative Competence Scale-Revised (C-PPCS-R34).

#### Confirmatory factor analysis

**Fig. 1:**
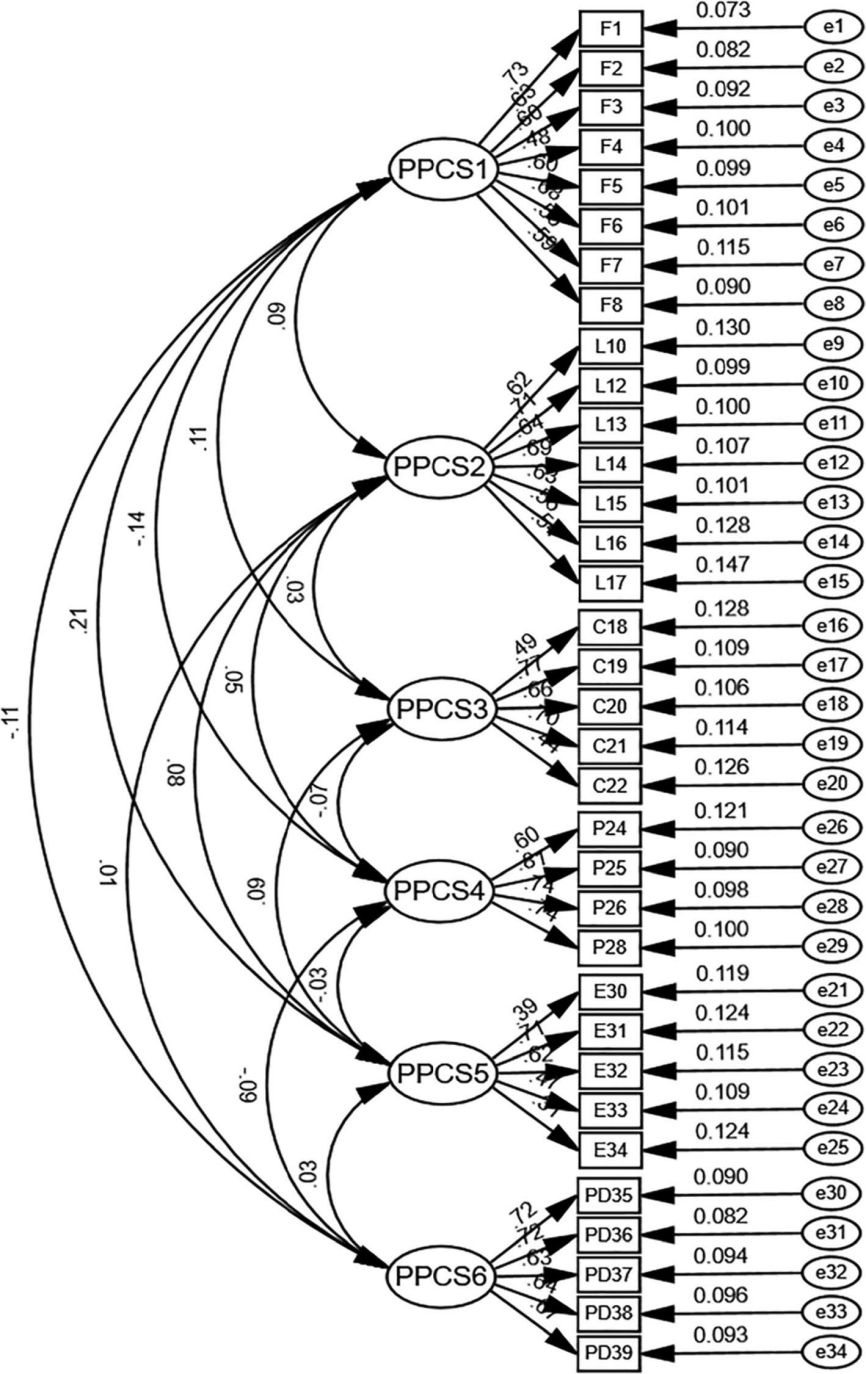
Confirmatory factor analysis results showing the standardized estimates with errors for the 34-item Chinese version of the Perceived Perioperative Competence Scale-Revised (C-PPCS-R34). F, Foundational knowledge and skills; L, Leadership; C, Collaboration; P, Proficiency; E, Empathy; PD, Professional Development

CFA (240 individuals, Group Two) with a robust maximum likelihood approximation was conducted to test the six-factor correlated model built on the basis of the EFA (240 individuals, Group One). The findings demonstrated a suitable fit: χ^2^ = 713.23, df = 513, *p* < 0.001; χ^2^/df = 1.39; RMSEA = 0.04; SRMR = 0.06; CFI = 0.91; and TLI = 0.90. Fig. 1 shows all standardized factor loadings.

#### Reliability analysis

The Cronbach’s alpha coefficient for the C-PPCS-R34 was reported 0.787 and the results for each of the six factors ranged from 0.792 to 0.949. More detail is provided in Table 6.

## Discussion

This article documents the translation, cultural adaption, and validation of the C-PPCS-R40 among operating room nurses in Beijing. This represents the first validation study of this scale in China. Our findings demonstrated that the C-PPCS-R34 was a valid and reliable evaluation measurement with adequate content validation, acceptable internal consistency, and good construct validation.

It is crucial that a scale model based on theory or previous analytical studies should be checked if it is used in a new setting [31]. To perform cross-cultural adaptations, researchers must have access to accurate and valid measurements of equivalent definitions in their own cultures and languages. A well-established methodology must also be accessible for the translation, adaptation and validation of instruments or scales for use in cross-cultural healthcare research [35]. In this study, it was used forward and back translation with five expert participants to develop the Chinese version of the scale. After discussion among these experts, some items in the original scale required modification as the meaning was repeated or the concepts were unclear in the Chinese context. For example, items 1 and 5 were related to familiar instrumentation and equipment, and items 35 and 37 were both related to maintaining current practical knowledge. Therefore, it was necessary to emphasize the differences between the items and provide examples in the revised version of the scale. The content validity of the C-PPCS-R40 indicated it was appropriate for the Chinese context. According to the item analysis, six items were identified for scale reduction. These items were selected through discrete trend analysis, factor analysis, the CR method, and the correlation coefficient method, all of which are classical test theory (CTT)-based screening methods. CTT, also known as true score theory, was the earliest test theory developed in the late 19th century and has been widely used. It assumes that the true score remains unchanged and any error is completely random. CTT is simple, easy to understand, has a complete system, and plays an important role in the selection of scale items [38]. The EFA and CFA results demonstrated good construct validity. The 34 retained items loaded on six factors that were named consistently with the original scale. Compared with the original scale, the variance contribution rate of all common factors decreased gradually: Foundational knowledge and skills (12.10%/15.52%), Leadership (11.10%/13.84%), Collaboration (9.20%/12.29%), Proficiency (9.00%/9.57%), Empathy (8.80%/9.26%), and Professional development (8.40%/8.14%) [25]. The contribution rate of variance corresponds to the proportion of the variation induced to the overall variation by a single common factor, which indicates the influence of that common factor on the dependent variable. The higher the variance rate, the stronger the influence of this factor [39]. The result indicated that the order of the importance of each competence in the original scale was suitable for the Chinese context. The CFA confirmed that the C-PPCS-R34 had a strong match, which corresponded closely to the versions used by Swedish OR nurses (SRMR = 0.07; RMSEA = 0.07) and Turkish OR nurses (SRMR = 0.07; RMSEA = 0.08) [31, 32]. The reliability (Cronbach’s alpha) of the 34-item scale was satisfactory. Finally, the new version of the scale was named the 34-item Chinese Perceived Perioperative Competence Scale-Revised (C-PPCS-R34).

The measurement structure of the C-PPCS-R34 is basically the same as for other language versions. Therefore, in subsequent applied research in China, the results can be compared with those for operating room nurses in other countries. The C-PPCS-R34 is a self-assessment tool that may assist in identifying and discussing nurses’ training needs and inform subsequent planning for training. Different career pathways in nursing require different competences. The C-PPCS-R34 may offer an evaluation method to support career planning based on nurses’ scores for the relevant subscales. In addition, using this scale in further research may facilitate exploration of the differences and degree of importance of competences in different job roles. It is also possible to examine the factors that influence differences in the competence of nurses in the operating room, whether nurses with higher qualifications, more years of experience or whether they have participated in specialist education are better equipped to work in the operating room. In previous studies from other countries, it has been demonstrated that specialist education and years of experience have an impact on OR competence [30]. Nurses’ own test results could provide them with references for their future career development.

### Limitations

Although we tried to ensure semantic equivalence in the process of translation, the author of the original PPCS-R was not included as a member of the panel for cross-cultural debugging. Therefore, we cannot guarantee that there was no deviation between the item-by-item semantics of the two scales. We suggest that future researchers should be cautious when using the C-PPCS-R34 in cross-cultural studies in the Chinese context.

This study was a multi-center cross-sectional study conducted across five third-grade class-A hospitals in Beijing and using a convenience sampling method. As a self-evaluation tool to assess operating room nurses’ perceived competency in China, the C-PPCS-R34 should be further validated in a broader multi-center cross-sectional study in other Chinese areas.

## Conclusions

The C-PPCS-R34 is a self-evaluation tool to assess operating room nurses’ perceived competence in China that has acceptable validity and reliability. In future applications, the assessment results may be quantified as the guiding basis for training of operating competences or to provide evaluation criteria for career planning and promotion evaluation of operating room nurses. Such evaluation is conducive to the development of the operating room nursing team and improvement of perioperative nursing abilities to ensure patient safety.

## Data Availability

The datasets generated and analyzed during the current study are not publicly available due the datum involve participant privacy concerns but are available from the corresponding author on reasonable request.

## Abbreviations

PPCS-R: Perceived Perioperative Competence Scale-Revised
C-PPCS-R: Chinese Perceived Perioperative Competence Scale-Revised
C-PPCS-R40: 40-item Chinese Perceived Perioperative Competence Scale-Revised
C-PPCS-R34: 34-item Chinese Perceived Perioperative Competence Scale-Revised
EFA: Exploratory Factor Analysis
CFA: Confirmatory Factor Analysis
I-CVI: Item Content Validity Index
S-CVI: Scale Content Validity Index
CV: Coefficient of Variation
CR: Critical Ratio
KMO: Kaiser-Meyer-Olkin test
SRMR: Standardized Root Mean Square Residual
RMSEA: Root Mean Square Error of Approximation
TLI: Tucker–Lewis index
CFI: Comparative Fit Index
C^2^: Communalities Values
